# Bis[1-(4-cyano­benz­yl)pyrazinium] bis­(1,2-dicyano­ethene-1,2-dithiol­ato)nickelate(II)

**DOI:** 10.1107/S1600536811022550

**Published:** 2011-06-18

**Authors:** Hui Zhang, Wen-Bo Pei, Shan-Shan Yu, Xiao-Ming Ren

**Affiliations:** a Department of Applied Chemistry, College of Science, Nanjing University of Technology, Nanjing 210009, People’s Republic of China; b School of Biochemical and Environmental Engineering, Nanjing Xiaozhuang College, Nanjing 210017, People’s Republic of China

## Abstract

The asymmetric unit of the title complex, (C_12_H_10_N_3_)_2_[Ni(C_4_N_2_S_2_)_2_], consists of one 1-(4-cyano­benz­yl)pyrazinium cation and one half of an [Ni(mnt)_2_]^2−^ dianion (mnt^2−^ is 1,2-dicyano­ethene-1,2-dithiol­ate). The Ni^2+^ ion is located on an inversion center and is coordinated by four S atoms from two mnt^2−^ ligands, exhibiting a square-planar coordination geometry. The cation adopts a conformation where both the pyrazine ring and the benzene ring are twisted with respect to the C—C—N reference plane by 16.5 (2) and 69.8 (1)°, respectively.

## Related literature

For general background to square-planar bis-1,2-dithiol­ato complexes of transition metals showing potential application as magnetic or conducting materials and other properties, see: Bigoli *et al.* (2002 ▶); Duan *et al.* (2010 ▶); Pei *et al.* (2011 ▶). For the synthesis, see: Davison & Holm (1967 ▶).
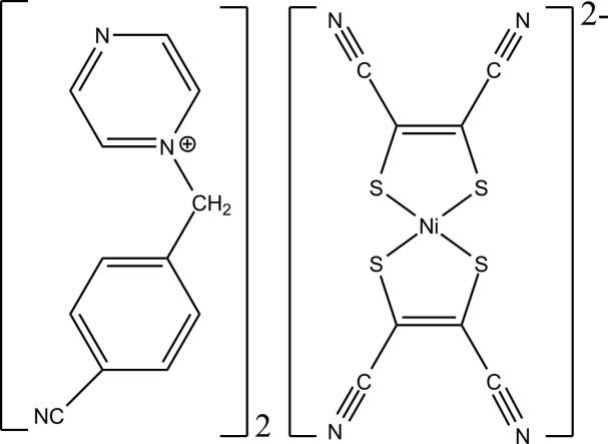


## Experimental

### 

#### Crystal data

(C_12_H_10_N_3_)_2_[Ni(C_4_N_2_S_2_)_2_]
*M* *_r_* = 731.53
Monoclinic, 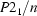

*a* = 7.115 (3) Å
*b* = 13.623 (6) Å
*c* = 17.186 (8) Å
β = 101.671 (5)°
*V* = 1631.4 (13) Å^3^
*Z* = 2
Mo *K*α radiation
μ = 0.89 mm^−1^
*T* = 298 K
0.30 × 0.20 × 0.15 mm

#### Data collection

Bruker SMART APEX CCD diffractometer
Absorption correction: multi-scan (*SADABS*; Sheldrick, 2002) ▶ *T*_min_ = 0.807, *T*_max_ = 0.875
8060 measured reflections
2871 independent reflections
2586 reflections with *I* > 2σ(*I*)
*R*_int_ = 0.020

#### Refinement

*R*[*F* ^2^ > 2σ(*F* ^2^)] = 0.026
*wR*(*F* ^2^) = 0.074
*S* = 1.06
2871 reflections
214 parameters
H-atom parameters constrained
Δρ_max_ = 0.21 e Å^−3^
Δρ_min_ = −0.26 e Å^−3^

### 

Data collection: *SMART* (Siemens, 1996 ▶); cell refinement: *SAINT* (Siemens, 1996 ▶); data reduction: *SAINT*; program(s) used to solve structure: *SHELXS97* (Sheldrick, 2008 ▶); program(s) used to refine structure: *SHELXL97* (Sheldrick, 2008 ▶); molecular graphics: *SHELXTL* (Sheldrick, 2008 ▶); software used to prepare material for publication: *SHELXTL*.

## Supplementary Material

Crystal structure: contains datablock(s) I, global. DOI: 10.1107/S1600536811022550/im2295sup1.cif

Structure factors: contains datablock(s) I. DOI: 10.1107/S1600536811022550/im2295Isup2.hkl

Additional supplementary materials:  crystallographic information; 3D view; checkCIF report

## Figures and Tables

**Fig. 1.**
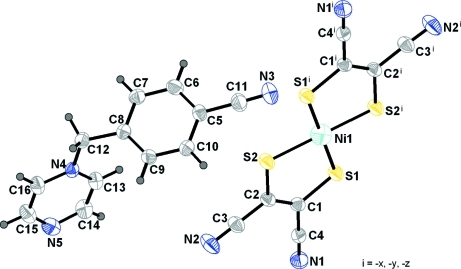
Molecular structure of (I). Displacement ellipsoids are drawn at the 30% probability level (i = -x, -y, -z).

**Fig. 2.**
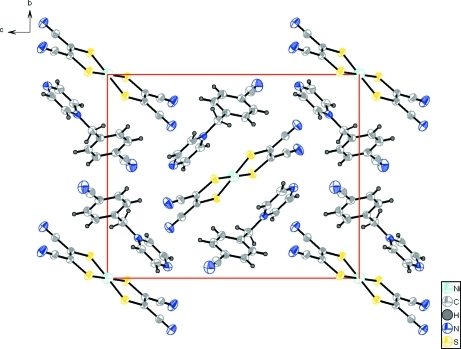
Packing diagram for (I) viewed along a axis.

## supplementary crystallographic information

### Comment

Bis-1,2-dithiolene complexes of transition metals have been widely studied because of their novel properties and applications in the areas of conducting and magnetic materials, dyes, nonlinear optics, catalysis and others. These applications arise due to a combination of functional properties, specific geometries and intermolecular interactions. (Bigoli *et al.*, 2002; Duan *et al.*, 2010; Pei *et al.*, 2011). Herein we report the crystal structure of the title compound (Fig. 1).

An asymmetric unit consists of one half [Ni(mnt)_2_]^2-^ dianion and one 1-*N*-(4'-cyano-benzyl)-pyrazinium cation. In the [Ni(mnt)_2_]^2-^ moiety, the Ni atom is situated at a inversion center and is coordinated by four S atoms from two mnt^2-^ ligands, forming a square-planar coordination geometry. The cation adopts a conformation where the bond lengths and bond angles were normal, and both the pyrazine ring and the phenyl ring are twisted with respect to the C8—C12—N4 reference plane with the corresponding dihedral angles of 16.5 (2)° and 69.8 (1)°.

### Experimental

Disodium maleonitriledithiolate (456 mg, 2.5 mmol) and nickel chloride hexahydrate (297 mg, 1.25 mmol) were mixed under stirring in water (20 ml) and heated to boiling for about 20 min. After the red solution was filtered an aqueous solution of 1-*N*-(4'-cyano-benzyl)-pyrazinium chloride (579 mg, 2.5 mmol) was added dropwise to the filtrate. The immediately formed dark red precipitate was filtered off, washed with water and dried in vacuum (yield: 722 mg, 79%). Block shaped single crystals suitable for X-ray analysis were obtained *via* recrystallization of the corresponding complex in acetone.

### Refinement

All non-hydrogen atoms were refined anisotropically, whereas the H atoms were calculated and placed to the bonded parent atoms in geometrically idealized positions (C—H = 0.93 or 0.97 Å) and refined as riding atoms, with *U*_iso_(H) = 1.2*U*_eq_(C).

### Crystal data

**Table:**

(C_12_H_10_N_3_)_2_[Ni(C_4_N_2_S_2_)_2_]	*F*(000) = 748
*M**_r_* = 731.53	*D*_x_ = 1.489 Mg m^−^^3^
Monoclinic, *P*2_1_/*n*	Melting point: 456 K
Hall symbol: -P 2yn	Mo *K*α radiation, λ = 0.71073 Å
*a* = 7.115 (3) Å	Cell parameters from 8060 reflections
*b* = 13.623 (6) Å	θ = 2.4–25.0°
*c* = 17.186 (8) Å	µ = 0.89 mm^−^^1^
β = 101.671 (5)°	*T* = 298 K
*V* = 1631.4 (13) Å^3^	Block, red
*Z* = 2	0.30 × 0.20 × 0.15 mm

### Data collection

**Table:**

Bruker SMART APEX CCD diffractometer	2871 independent reflections
Radiation source: fine-focus sealed tube	2586 reflections with *I* > 2σ(*I*)
graphite	*R*_int_ = 0.020
φ and ω scans	θ_max_ = 25.0°, θ_min_ = 2.4°
Absorption correction: multi-scan (*SADABS*; Sheldrick, 2002)	*h* = −8→4
*T*_min_ = 0.807, *T*_max_ = 0.875	*k* = −16→15
8060 measured reflections	*l* = −20→20

### Refinement

**Table:**

Refinement on *F*^2^	Primary atom site location: structure-invariant direct methods
Least-squares matrix: full	Secondary atom site location: difference Fourier map
*R*[*F*^2^ > 2σ(*F*^2^)] = 0.026	Hydrogen site location: inferred from neighbouring sites
*wR*(*F*^2^) = 0.074	H-atom parameters constrained
*S* = 1.06	*w* = 1/[σ^2^(*F*_o_^2^) + (0.0431*P*)^2^ + 0.3009*P*] where *P* = (*F*_o_^2^ + 2*F*_c_^2^)/3
2871 reflections	(Δ/σ)_max_ < 0.001
214 parameters	Δρ_max_ = 0.21 e Å^−^^3^
0 restraints	Δρ_min_ = −0.26 e Å^−^^3^

### Special details

**Table:**

Geometry. All e.s.d.'s (except the e.s.d. in the dihedral angle between two l.s. planes) are estimated using the full covariance matrix. The cell e.s.d.'s are taken into account individually in the estimation of e.s.d.'s in distances, angles and torsion angles; correlations between e.s.d.'s in cell parameters are only used when they are defined by crystal symmetry. An approximate (isotropic) treatment of cell e.s.d.'s is used for estimating e.s.d.'s involving l.s. planes.
Refinement. Refinement of *F*^2^ against ALL reflections. The weighted *R*-factor *wR* and goodness of fit *S* are based on *F*^2^, conventional *R*-factors *R* are based on *F*, with *F* set to zero for negative *F*^2^. The threshold expression of *F*^2^ > σ(*F*^2^) is used only for calculating *R*-factors(gt) *etc*. and is not relevant to the choice of reflections for refinement. *R*-factors based on *F*^2^ are statistically about twice as large as those based on *F*, and *R*-factors based on ALL data will be even larger.

### Fractional atomic coordinates and isotropic or equivalent isotropic displacement parameters (Å^2^)

**Table:**

	*x*	*y*	*z*	*U*_iso_*/*U*_eq_	
Ni1	0.0000	0.0000	0.0000	0.04070 (11)	
C1	0.0668 (2)	0.13286 (12)	0.14603 (9)	0.0452 (4)	
C2	0.2425 (2)	0.09233 (12)	0.14864 (10)	0.0464 (4)	
C3	0.4020 (3)	0.11475 (13)	0.21086 (11)	0.0566 (4)	
C4	0.0334 (2)	0.19910 (13)	0.20637 (10)	0.0508 (4)	
C5	0.3059 (2)	0.61780 (13)	−0.03334 (11)	0.0515 (4)	
C6	0.4014 (3)	0.67295 (15)	−0.08013 (11)	0.0608 (5)	
H6A	0.3440	0.6865	−0.1326	0.073*	
C7	0.5826 (3)	0.70790 (15)	−0.04870 (11)	0.0594 (5)	
H7A	0.6473	0.7448	−0.0805	0.071*	
C8	0.6697 (2)	0.68900 (12)	0.02927 (10)	0.0473 (4)	
C9	0.5730 (2)	0.63300 (13)	0.07578 (10)	0.0513 (4)	
H9A	0.6311	0.6190	0.1281	0.062*	
C10	0.3910 (3)	0.59771 (13)	0.04520 (11)	0.0550 (4)	
H10A	0.3260	0.5608	0.0769	0.066*	
C11	0.1164 (3)	0.58128 (16)	−0.06742 (13)	0.0670 (5)	
C12	0.8708 (2)	0.72452 (14)	0.06029 (11)	0.0541 (4)	
H12A	0.9486	0.6693	0.0833	0.065*	
H12B	0.9232	0.7484	0.0159	0.065*	
C13	0.7384 (3)	0.85735 (13)	0.13272 (12)	0.0558 (4)	
H13A	0.6171	0.8476	0.1014	0.067*	
C14	0.7677 (3)	0.92685 (15)	0.19170 (13)	0.0672 (5)	
H14A	0.6632	0.9643	0.1989	0.081*	
C15	1.0821 (3)	0.89226 (14)	0.22341 (12)	0.0658 (5)	
H15A	1.2038	0.9037	0.2539	0.079*	
C16	1.0618 (3)	0.82300 (14)	0.16465 (11)	0.0573 (5)	
H16A	1.1684	0.7893	0.1549	0.069*	
N1	0.0083 (2)	0.25233 (14)	0.25432 (10)	0.0707 (5)	
N2	0.5329 (3)	0.13221 (16)	0.25944 (11)	0.0842 (6)	
N3	−0.0327 (3)	0.55373 (19)	−0.09570 (13)	0.0963 (7)	
N4	0.88726 (18)	0.80422 (10)	0.12132 (8)	0.0455 (3)	
N5	0.9357 (3)	0.94368 (12)	0.23882 (10)	0.0677 (4)	
S1	−0.12375 (6)	0.10653 (3)	0.06901 (3)	0.05089 (13)	
S2	0.28315 (7)	0.01308 (3)	0.07488 (3)	0.05495 (14)	

### Atomic displacement parameters (Å^2^)

**Table:**

	*U*^11^	*U*^22^	*U*^33^	*U*^12^	*U*^13^	*U*^23^
Ni1	0.04532 (18)	0.03877 (17)	0.03648 (17)	−0.00199 (11)	0.00463 (12)	−0.00037 (10)
C1	0.0529 (9)	0.0417 (8)	0.0406 (8)	−0.0064 (7)	0.0085 (7)	−0.0019 (7)
C2	0.0511 (9)	0.0440 (8)	0.0423 (8)	−0.0041 (7)	0.0051 (7)	−0.0038 (7)
C3	0.0564 (11)	0.0559 (10)	0.0541 (10)	0.0060 (8)	0.0036 (9)	−0.0140 (8)
C4	0.0470 (9)	0.0544 (10)	0.0508 (9)	−0.0046 (7)	0.0094 (8)	−0.0047 (8)
C5	0.0449 (9)	0.0502 (9)	0.0585 (10)	0.0017 (7)	0.0079 (8)	−0.0081 (8)
C6	0.0608 (11)	0.0711 (12)	0.0473 (10)	0.0045 (9)	0.0033 (8)	0.0033 (9)
C7	0.0599 (11)	0.0661 (11)	0.0531 (10)	−0.0045 (9)	0.0135 (8)	0.0121 (9)
C8	0.0461 (9)	0.0454 (9)	0.0512 (9)	−0.0015 (7)	0.0118 (7)	0.0002 (7)
C9	0.0520 (10)	0.0547 (10)	0.0463 (9)	−0.0037 (8)	0.0074 (7)	0.0036 (7)
C10	0.0533 (10)	0.0555 (10)	0.0577 (10)	−0.0076 (8)	0.0147 (8)	0.0039 (8)
C11	0.0542 (12)	0.0750 (13)	0.0690 (13)	−0.0025 (10)	0.0059 (10)	−0.0086 (10)
C12	0.0479 (9)	0.0575 (10)	0.0587 (10)	−0.0046 (8)	0.0153 (8)	−0.0027 (8)
C13	0.0464 (9)	0.0524 (10)	0.0664 (11)	−0.0008 (8)	0.0066 (8)	0.0011 (8)
C14	0.0634 (12)	0.0577 (11)	0.0791 (14)	0.0017 (9)	0.0111 (10)	−0.0062 (10)
C15	0.0651 (12)	0.0559 (11)	0.0669 (12)	−0.0098 (9)	−0.0091 (10)	0.0106 (9)
C16	0.0455 (9)	0.0546 (10)	0.0673 (11)	−0.0028 (8)	0.0006 (8)	0.0099 (9)
N1	0.0665 (11)	0.0800 (12)	0.0676 (11)	0.0002 (9)	0.0182 (8)	−0.0250 (9)
N2	0.0671 (11)	0.0967 (14)	0.0773 (12)	0.0143 (10)	−0.0129 (10)	−0.0363 (11)
N3	0.0612 (12)	0.1261 (19)	0.0940 (15)	−0.0216 (12)	−0.0021 (11)	−0.0063 (14)
N4	0.0427 (7)	0.0434 (7)	0.0499 (8)	−0.0049 (6)	0.0080 (6)	0.0103 (6)
N5	0.0823 (12)	0.0551 (9)	0.0618 (10)	−0.0071 (9)	0.0051 (9)	0.0015 (8)
S1	0.0477 (2)	0.0538 (3)	0.0488 (2)	0.00206 (18)	0.00416 (18)	−0.00887 (18)
S2	0.0479 (3)	0.0615 (3)	0.0517 (3)	0.00499 (19)	0.0009 (2)	−0.01617 (19)

### Geometric parameters (Å, °)

**Table:**

Ni1—S2	2.1674 (9)	C8—C12	1.502 (2)
Ni1—S2^i^	2.1674 (9)	C9—C10	1.382 (2)
Ni1—S1	2.1704 (7)	C9—H9A	0.9300
Ni1—S1^i^	2.1704 (7)	C10—H10A	0.9300
C1—C2	1.359 (2)	C11—N3	1.138 (3)
C1—C4	1.430 (2)	C12—N4	1.498 (2)
C1—S1	1.7302 (17)	C12—H12A	0.9700
C2—C3	1.426 (2)	C12—H12B	0.9700
C2—S2	1.7334 (18)	C13—N4	1.330 (2)
C3—N2	1.143 (2)	C13—C14	1.372 (3)
C4—N1	1.139 (2)	C13—H13A	0.9300
C5—C6	1.376 (3)	C14—N5	1.322 (3)
C5—C10	1.390 (3)	C14—H14A	0.9300
C5—C11	1.445 (3)	C15—N5	1.325 (3)
C6—C7	1.378 (3)	C15—C16	1.368 (3)
C6—H6A	0.9300	C15—H15A	0.9300
C7—C8	1.382 (2)	C16—N4	1.337 (2)
C7—H7A	0.9300	C16—H16A	0.9300
C8—C9	1.384 (2)		
			
S2—Ni1—S2^i^	180.00 (2)	C9—C10—C5	119.44 (17)
S2—Ni1—S1	92.96 (3)	C9—C10—H10A	120.3
S2^i^—Ni1—S1	87.04 (3)	C5—C10—H10A	120.3
S2—Ni1—S1^i^	87.04 (3)	N3—C11—C5	178.5 (3)
S2^i^—Ni1—S1^i^	92.96 (3)	N4—C12—C8	114.59 (14)
S1—Ni1—S1^i^	180.00 (2)	N4—C12—H12A	108.6
C2—C1—C4	121.29 (15)	C8—C12—H12A	108.6
C2—C1—S1	120.95 (13)	N4—C12—H12B	108.6
C4—C1—S1	117.76 (13)	C8—C12—H12B	108.6
C1—C2—C3	121.67 (15)	H12A—C12—H12B	107.6
C1—C2—S2	121.17 (12)	N4—C13—C14	118.57 (17)
C3—C2—S2	117.14 (13)	N4—C13—H13A	120.7
N2—C3—C2	178.3 (2)	C14—C13—H13A	120.7
N1—C4—C1	179.3 (2)	N5—C14—C13	123.76 (19)
C6—C5—C10	120.33 (16)	N5—C14—H14A	118.1
C6—C5—C11	118.78 (17)	C13—C14—H14A	118.1
C10—C5—C11	120.88 (17)	N5—C15—C16	122.83 (19)
C5—C6—C7	119.54 (17)	N5—C15—H15A	118.6
C5—C6—H6A	120.2	C16—C15—H15A	118.6
C7—C6—H6A	120.2	N4—C16—C15	119.37 (18)
C6—C7—C8	121.07 (17)	N4—C16—H16A	120.3
C6—C7—H7A	119.5	C15—C16—H16A	120.3
C8—C7—H7A	119.5	C13—N4—C16	119.44 (16)
C7—C8—C9	119.00 (16)	C13—N4—C12	123.08 (14)
C7—C8—C12	119.48 (16)	C16—N4—C12	117.47 (15)
C9—C8—C12	121.42 (16)	C14—N5—C15	115.85 (18)
C10—C9—C8	120.61 (16)	C1—S1—Ni1	102.41 (7)
C10—C9—H9A	119.7	C2—S2—Ni1	102.28 (6)
C8—C9—H9A	119.7		

Symmetry codes: (i) −*x*, −*y*, −*z*.
